# Peak Resembling N-acetylaspartate (NAA) on Magnetic Resonance Spectroscopy of Brain Metastases

**DOI:** 10.3390/medicina60040662

**Published:** 2024-04-19

**Authors:** Jelena Ostojic, Dusko Kozic, Milana Panjkovic, Biljana Georgievski-Brkic, Dusan Dragicevic, Aleksandra Lovrenski, Jasmina Boban

**Affiliations:** 1 Faculty of Medicine, University in Novi Sad, 21000 Novi Sad, Serbia; dusko.kozic@mf.uns.ac.rs (D.K.); milana.panjkovic@mf.uns.ac.rs (M.P.); dragicevic.dusan@onk.ns.ac.rs (D.D.); aleksandra.lovrenski@mf.uns.ac.rs (A.L.); jasmina.boban@mf.uns.ac.rs (J.B.); 2 Special Hospital for Cerebrovascular Diseases “Sveti Sava”, 11000 Belgrade, Serbia; radiologija@svetisava.rs

**Keywords:** magnetic resonance spectroscopy, brain, neoplasm, metastasis, differential diagnosis, mucin

## Abstract

*Background and Objectives*: Differentiating between a high-grade glioma (HGG) and solitary cerebral metastasis presents a challenge when using standard magnetic resonance imaging (MRI) alone. Magnetic resonance spectroscopy (MRS), an advanced MRI technique, may assist in resolving this diagnostic dilemma. N-acetylaspartate (NAA), an amino acid found uniquely in the central nervous system and in high concentrations in neurons, typically suggests HGG over metastatic lesions in spectra from ring-enhancing lesions. This study investigates exceptions to this norm. *Materials and Methods*: We conducted an MRS study on 49 histologically confirmed and previously untreated patients with brain metastases, employing single-voxel (SVS) techniques with short and long echo times, as well as magnetic resonance spectroscopic imaging (MRSI). *Results:* In our cohort, 44 out of 49 (90%) patients demonstrated a typical MR spectroscopic profile consistent with secondary deposits: a Cho peak, very low or absent Cr, absence of NAA, and the presence of lipids. A peak at approximately 2 ppm, termed the “NAA-like peak”, was present in spectra obtained with both short and long echo times. Among the MRS data from 49 individuals, we observed a peak at 2.0 ppm in five brain metastases from mucinous carcinoma of the breast, mucinous non-small-cell lung adenocarcinoma, two metastatic melanomas, and one metastatic non-small-cell lung cancer. Pathohistological verification of mucin in two of these five cases suggested this peak likely represents N-acetyl glycoproteins, indicative of mucin expression in cancer cells. *Conclusions:* The identification of a prominent peak at 2.0 ppm could be a valuable diagnostic marker for distinguishing single ring-enhancing lesions, potentially associated with mucin-expressing metastases, offering a new avenue for diagnostic specificity in challenging cases.

## 1. Introduction

Discriminating between an HGG and a solitary brain metastasis remains a significant challenge for neuroradiologists relying solely on standard magnetic resonance imaging (MRI). Accurate diagnosis in such cases is pivotal for guiding subsequent diagnostic and therapeutic interventions [1,2]. Multiparametric MRI, which integrates conventional protocols with diffusion-weighted imaging (DWI), magnetic resonance spectroscopy (MRS), and dynamic susceptibility contrast imaging (DSCI), has emerged as a valuable tool for discerning between HGGs and solitary metastatic brain lesions [3,4]. MRS is used to obtain neurometabolic data from a specific volume of interest in the brain parenchyma. The following metabolites are the most commonly analyzed in the context of metastasis: choline (Cho), creatine (Cr), NAA, lipids, and lactate [5]. Cho, a vital component of cell membranes, resonates prominently at approximately 3.2 ppm, offering crucial insight into cellular membrane dynamics, cell signaling, and lipid metabolism. Elevated Cho levels, detected by MRS, have been observed in various cancers, including breast, prostate, brain, and ovarian cancers, indicating aberrant choline metabolism, a hallmark of malignant cells. In metastasis, heightened Cho peaks suggest increased membrane turnover and cell proliferation, characteristic of aggressive tumor behavior [6,7]. The elevation of Cho-containing compounds, such as phosphocholine and glycerophosphocholine, reflects increased cell membrane synthesis and breakdown, as well as alterations in phospholipid metabolism, which are often associated with tumor growth and invasion [7,8]. The Cr peak in brain MRS, resonating at 3 ppm, provides valuable information about cellular energy status and metabolism in the brain. Cr, predominantly found in neurons and glial cells, plays a crucial role in energy metabolism by serving as a reservoir for high-energy phosphate bonds [9]. Previous studies have shown that the presence of intratumoral Cr is indicative of glioma, whereas its absence suggests metastasis [10,11]. Heightened lipid signals observed in MRS often serve as indicators of underlying pathological processes, including necrosis, inflammation, or demyelination. Increases in lipid levels typically arise from the breakdown of cell membranes and myelin sheaths, leading to the release of lipid constituents into the extracellular milieu. In brain MRS, these signals emanate from mobile lipids, encompassing fatty acids and triglycerides, which contain characteristic chemical moieties, such as CH2 (methylene) and CH3 (methyl). Notably, a significant increase in lipid content detected via MRS is commonly recognized as a hallmark feature of brain metastases [12,13]. NAA, primarily localized in neurons, serves as a marker for neuronal viability, synaptic health, and metabolism, resonating at approximately 2.0 ppm on MR spectroscopy. Alterations in NAA levels detected through MRS have been documented in brain tumors, including metastases, indicating shifts in neuronal integrity and metabolism. Reduced NAA levels often correlate with tumor infiltration into healthy brain tissue, neuronal decline, and compromised neuronal function [14]. Metastases typically lack NAA signals in both in vitro and in vivo studies, with any presence likely attributed to contamination by normal brain tissue [15,16,17]. Therefore, the absence of NAA is an important diagnostic criterion suggesting that an intracranial mass lesion is a metastasis. The presence of a peak resembling NAA in the spectrum of neoplastic brain lesions may be interpreted as a sign of infiltration, indicative of a high-grade infiltrative tumor, in clinical practice. This interpretation could significantly impact the timely administration of appropriate therapy. Therefore, this study aims to present the occurrence of a prominent peak at approximately 2 ppm, mimicking NAA, in histologically proven solid metastatic brain lesions. Additionally, our objective is to contribute to the understanding of the properties of this peak, which likely represents a component of mucin. The role of mucin in carcinomas is not fully understood and remains an area of intense research.

## 2. Materials and Methods

We conducted a magnetic resonance spectroscopy (MRS) study, approved by the ethics board, involving 75 previously untreated patients diagnosed with brain metastases. This study was conducted as part of a preoperative clinical assessment preceding surgery, stereotactic biopsy, or stereotactic radiosurgery. Since treatment was not administered at the institution where diagnostic examinations were conducted, we were able to obtain histopathological confirmations for 49 brain metastases. The characteristics of the participant group, including the number of patients with brain metastases by the organs from which the primary tumor originated, median age, and gender, are provided in Table 1. All patients were of Caucasian race. Since pathology does not routinely provide reports on the presence of mucin in metastases, we were able to retrospectively obtain confirmation of mucin presence for two out of five metastases in the group exhibiting unusual peaks at 2 ppm. For 48 patients, a routine brain MRI protocol with contrast was completed prior to MRS, consisting of Sagittal T1-weighted spin-echo (SE) sequences with a repetition time/echo time (TR/TE) of 550/8.7 ms and 5 mm thickness, axial T2-weighted turbo SE (TSE) with a TR/TE of 4930/114 ms and 5 mm thickness, axial T2* with a TR/TE of 857/26 ms, and coronal T2 TSE with a TR/TE of 5530/88 ms and 5 mm thickness. Axial Fluid Attenuated Inversion Recovery (FLAIR) was conducted with a TR/TE of 9000/92 ms and 5 mm thickness, axial diffusion with a TR/TE of 5348/102 and 5 mm thickness, pre- and post-contrast axial T1 SE with a TR/TE of 550/8.7, and post-contrast 3D T1 with a TR/TE of 2200/2.8 ms. For 1 patient, a routine 3T brain MRI protocol with contrast was completed prior to MRS, consisting of Sagittal T1-weighted SE sequences (TR/TE: 251/3.8 ms, thickness: 5 mm), axial T2-weighted TSE with a TR/TE of 5750/114 ms and a thickness of 5 mm, Axial FLAIR with a TR/TE of 9000/97 ms and a thickness of 5 mm, axial diffusion with a TR/TE of 4000/91 ms and a thickness of 5 mm, 3D susceptibility-weighted imaging (SWI) with a TR/TE of 29/20 ms and a thickness of 2.5 mm, pre- and post-contrast axial T1 SE with a TR/TE of 175/2.7 ms, and post-contrast 3D T1 with a TR/TE of 1900/2.3 ms and a thickness of 1 mm. In certain clinical situations, depending on the need, for better visualization, we added sequences such as T2 FLAIR in the coronal orientation. Single-voxel point resolved spectroscopy sequence (PRESS) with repetition time/echo times (TR/TE) of 1500/30 or 1500/35 ms and TR/TE of 1500/135 or 1500/144 ms (depending on whether we used a Siemens or GE MR unit; the first value refers to Siemens, and the second to GE), were performed on all patients using a 1.5T MRI unit (Magnetom-Aera; Siemens, Erlangen, Germany or Signa HDxT GE Healthcare, Milwaukee, WI, USA). All localizations were verified by an experienced neuroradiologist. In most cases, the SVS volume of interest (VOI) size was 15 × 15 × 15 mm^3^, although, in some instances, we adjusted the VOI dimensions to match the size of the metastasis. We performed MRS only on metastases larger than 12 mm in diameter. The VOI size and position were determined using MR images in all three dimensions (axial, sagittal, and coronal). If required, necrotic or cystic areas within tumors were encompassed within the voxels to minimize contamination from normal brain tissue. Each SVS spectrum was obtained as an average of 128 measurements. A total of 98 SVS spectra were analyzed in this study, 7 of which were discarded due to poorer technical quality. Despite that MRS was performed exclusively on metastases exceeding 12 mm in diameter, it was not possible to apply the same VOI size to all patients, due to the varying dimensions of their metastases. We always endeavored to position the SVS VOI precisely within the lesion and to minimize the inclusion of surrounding tissue in the voxel under examination. As an additional measure, in patients with good general condition who consented to longer scans (30 from 49 patients in our group), we conducted two-dimensional (2D) or three-dimensional (3D) multi-voxel PRESS or MR spectroscopic imaging (MRSI) to assess the spatial distribution of metabolites in metastases and evaluate surrounding edema. For 2D MRSI, the TR/TE was 1500/135 or 1500/144 ms, and the field of view (FOV) was 160 × 160 × 160 mm^3^, with a VOI size of 80 × 80 × 80 mm^3^ and a thickness of 10 mm. The number of phase-encoding steps (scan resolution) was 16 in all directions (R-L, A-P, and F-H), and the number of reconstructed spectra (interpolation resolution) was 16 in all directions, resulting in a VOI size of 10 × 10 × 10 mm^3^. When 3D MRSI PRESS was applied, TR/TE was 1500/135 or 1500/144 ms. The MRSI slab size, with a FOV of 80 × 80 × 80 mm^3^ and a VOI of 40 × 40 × 40 mm^3^, was positioned parallel to the axial images, covering the metastasis and surrounding edema. The number of phase-encoding steps was 12 in the R–L and A–P directions and 8 in the F–H direction, resulting in a VOI of 6.67 × 6.67 × 10 mm^3^. We utilized a 3T MRI unit (Trio Siemens) for only one patient (Patient 1). The only difference in MRS parameters compared to the described 1.5T protocols was a longer TR time: TR/TE = 1700/135 and TR/TE = 1700/135 for SVS spectroscopy; MRSI was not performed in this patient. Multiple rest slabs were manually positioned along the margin of each VOI to prevent contamination of spectral signals from subcutaneous fat. The MRSI raw data were evaluated using commercially available spectral analysis software packages (Syngo Multi ModalityWorkplace, version VE23 A, Siemens, Erlangen, Germany; or General Electric Functool Advantage 4.7, GE Medical System/Healthcare, Waukesha, WI, USA). The post-processing protocol included water reference processing by averaging 20 adjacent points, removing the residual water signal from the spectrum by subtracting it from the time signal and frequency shift correction of the water signal, using a Hanning filter with 512 ms width, zero filling from 512 to 1024 data points, and Fourier transform. Signals for Cr (3.04 ppm), Cho (3.2 ppm), NAA (2.02 ppm), CH2 (1.3 ppm), and CH3 (at 0.9 ppm) lipids were evaluated using Gaussian curve fittings. Due to very low or completely absent Cr and NAA in metastases, when there is no contamination with signals from surrounding structures, we were unable to calculate the relative concentrations of Cho compared to Cr or NAA. We investigated the presence or absence of the peak recognized by the curve-fitting program as NAA in the spectra of metastases.

## 3. Results

**Figure 1:**
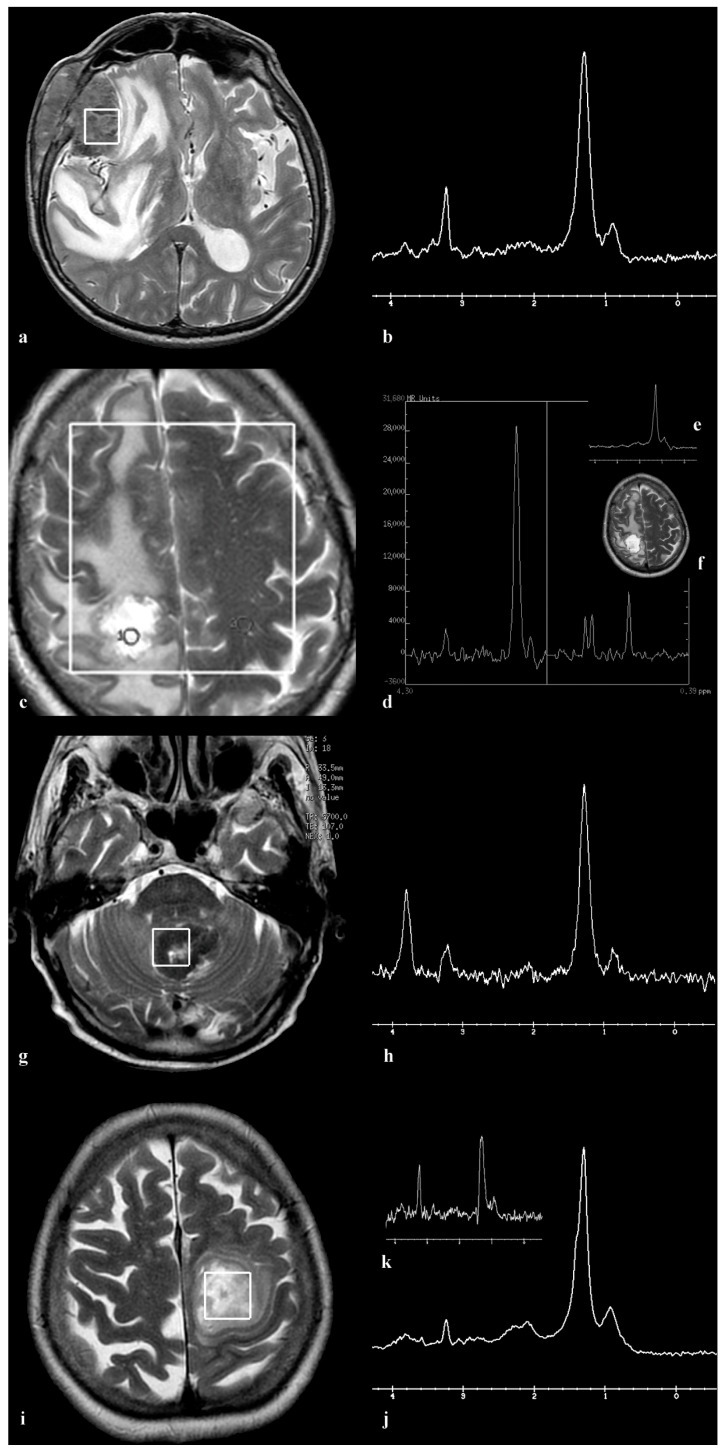
This series of images showcases typical findings on magnetic resonance spectroscopy (MRS) in brain metastases originating from various primary cancers. (**a**) illustrates the placement of a VOI in a metastasis from prostate adenocarcinoma. This metastasis is presented as a T2 hypointense lesion in the right frontal–temporal region, causing frontal bone destruction and scalp tissue penetration, accompanied by extensive perifocal edema. (**b**) presents an SVS spectrum at an echo time (TE) of 144 ms, with notable Cho peak at 3.2 ppm, a distinct lipid CH2 signal at 1.2 ppm, and a lower signal of CH3 lipids at 0.9 ppm. (**c**) shows a metastasis from lung squamous cell carcinoma located in the upper right parasagittal frontal gyrus, characterized by extensive edema. It also shows the MRSI TE = 144 volume covering both hemispheres and targeted regions. (**d**) reveals the lesion’s spectrum, highlighting Cho and lipid peaks, contrasted with normal tissue in the left hemisphere. (**e**) displays the SVS TE = 30 ms spectrum of the same patient, featuring lipid peaks with a prominent CH2 and a low CH3 component. The position of SVS in the center of the lesion is shown in (**f**). (**g**) details a bilateral expansive lesion in the retrocerebellar and vermian regions, displaying a heterogeneous signal intensity decrease on T1/T2/FLAIR. Histopathological examination confirmed lung adenocarcinoma metastasis. (**h**) shows the TE = 144 ms spectrum, including Cho, high CH2, low CH3 lipid peaks, and a mannitol peak. (**i**) features an expansive high frontal parasagittal lesion on the left, affecting the centrum semiovale with mixed signal intensities and necrosis zones. The biopsy indicated a metastasis originating from invasive ductal carcinoma of the breast. The voxel positioning for SVS spectroscopy, consistent across TE = 30 ms (**j**) and TE = 144 ms (**k**) spectra, demonstrates predominant Cho and lipid signals with a leading CH2 component.

In our study, involving 49 cases of brain metastases, 44 (90%) patients demonstrated a typical MR spectroscopic profile consistent with secondary deposits: a choline (Cho) peak, very low or absent creatine (Cr), absence of N-acetylaspartate (NAA), and the presence of CH2 and CH3 lipids, with a predominant CH2 component (Figure 1). Similar findings were obtained using both short and long echo time spectroscopy, though with lower lipid and higher choline peak amplitudes observed in spectra acquired with longer echo times (TE = 135 or 144 ms). In five patients (10%), each with a known primary tumor—including mucinous carcinoma of the breast, mucinous non-small-cell lung adenocarcinoma, two cases of metastatic melanoma, and one case of metastatic non-small-cell lung cancer—we identified a high peak around 2.0 ppm, recognized by the curve-fitting program as NAA. This peak was distinctly observable in spectra obtained with both short and long echo times, with lower amplitude in spectra acquired with longer echo times (TE = 135 or 144 ms) compared to those acquired with shorter echo times (TE = 30 or 35 ms). Using MRSI, we observed a high degree of homogeneity in brain metastases, with spectra showing consistency throughout all regions of the lesion, except for cystic and necrotic areas. The greatest deviations were observed in the amplitudes of lipid peaks. This uniformity was evident in both typical metastases and those displaying an unusual peak around 2 ppm, which consistently appeared in all spectra of the lesion, regardless of the voxel position within the lesion.

### 3.1. Patient 1

**Figure 2:**
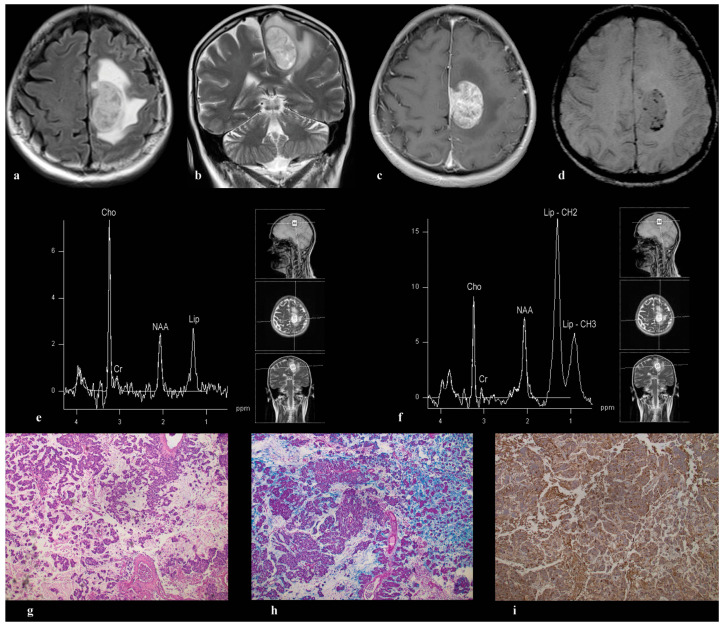
A large oval-shaped lesion, observed in a female patient, aged 62, expanding parasagittally in the frontoparietal region of the left hemisphere and adherent to the dura, is depicted in (**a**) (Axial FLAIR) and (**b**) (Coronal T2). Surrounding this lesion is perifocal edema. The T1 post-contrast SE image (**c**) reveals intense contrast enhancement within the lesion, and signs of microhemorrhage on SWI (**d**) are evident. Analysis of the single-voxel MRS with TE = 135 ms demonstrates elevated choline and NAA-like peaks at approximately 2.0 ppm (**e**), accompanied by a prominent lipid peak at 1.3 ppm (**f**). The signal intensity of the peak around 2 ppm is notably higher in the short echo spectrum, along with a peak at approximately 3.8 ppm (**f**). Immunohistochemical analysis, using antibodies including Anti-OLIG2, progesterone, estrogen, cytokeratin 7, cytokeratin 20, and mammaglobin, confirms the diagnosis of metastatic mucinous breast carcinoma (**g**). Furthermore, Alcian blue histochemical staining demonstrates mucin predominantly in the extracellular space (blue staining) (**h**), while immunohistochemical staining with anti MUC1 antibody highlights membranous staining of tumor cells (**i**).

A 62-year-old female presented with a large oval-shaped frontoparietal parasagittal expansive lesion in the left hemisphere, attached to the dura, surrounded by extensive perifocal edema (Figure 2a,b). Following the administration of contrast, a pronounced and heterogeneous enhancement pattern was noted (Figure 2c), with blood components detected within (Figure 2d). The voxels for MRS were centrally located within the enhancing mass in the left frontal lobe. Spectra obtained at both echo times, TE = 135 ms (Figure 2e) and TE = 30 ms (Figure 2f) revealed a single peak near 2 ppm resembling NAA, accompanied by elevated Cho, diminished resonance from Cr, and lipids. Routine pathohistological examination was performed after surgery using standard procedures and Hematoxylin–eosin (HE) staining, as well as immunohistochemical staining, to distinguish a primary brain tumor from brain metastasis of breast carcinoma. Anti-OLIG2, progesterone, estrogen, cytokeratin 7, cytokeratin 20, and mammaglobin antibodies were used, and the diagnosis of metastatic mucinous breast carcinoma was established (Figure 2g). Alcian blue histochemical staining was used to confirm the presence of mucin, dominantly in the extracellular space (blue staining) (Figure 2h), while immunohistochemical staining with anti MUC1 antibody was used for the same purpose (membranous staining of tumor cells) (Figure 2i).

### 3.2. Patient 2

**Figure 3:**
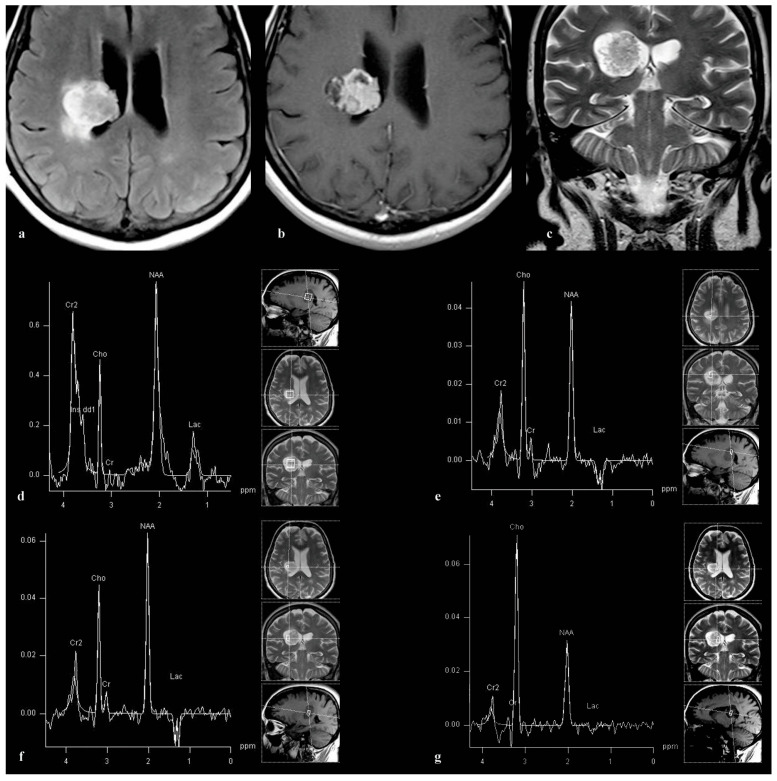
A heterogeneous subependymal lesion of expansive nature is observed in a 55-year-old female patient, partially extending into the body of the left lateral ventricle, as depicted in axial FLAIR (**a**), axial post-contrast T1 SE (**b**), and coronal TSE T2 (**c**). The lesion exhibits intense contrast enhancement with minimal associated edema. Analysis of the single-voxel MRS spectrum with TE = 30 ms reveals a prominent NAA-like peak at 2.0 ppm, alongside lactate and a substantial component at approximately 3.8 ppm (**d**). Employing 3D multi-voxel spectroscopy with TE = 135 ms, the entire lesion was assessed, with the ≈2.05 ppm peak consistently observed across all spectra within the lesion. (**e**–**g**) illustrate three voxels from a 3D multi-voxel volume. Surgical biopsy of the lesion confirmed a diagnosis of melanoma metastasis.

A 55-year-old female exhibited a focal expansive lesion situated in the subependymal periventricular region on the left side, characterized by prominent contrast enhancement and minimal edema (Figure 3a–c). Both short (Figure 3d) and long echo time spectroscopy (Figure 3e–g) revealed a prominent peak around 2 ppm, lactate, high Cho, and very low Cr. The short echo spectrum (TE = 30 ms) exhibited a significant component at approximately 3.8 ppm (Figure 3d), indicative of sialic acid. A surgical biopsy of the lesion was performed, revealing a diagnosis of melanoma metastasis.

### 3.3. Patient 3

**Figure 4:**
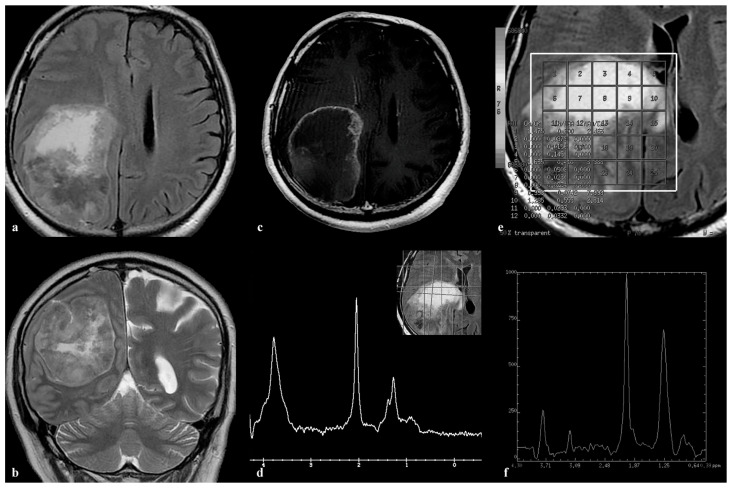
A notable lesion observed in a 58-year-old male patient, characterized by central necrosis and expansive features, is seen within the right parietal lobe, as depicted in the axial FLAIR image (**a**) and coronal FLAIR image (**b**). Surrounding edema is minimal, displaying a distinct ring-enhancing pattern, as shown in (**c**). Analysis of SVS with TE = 35 ms demonstrates a pronounced narrow peak at approximately 2.05 ppm, accompanied by a lipid peak at 1.3 ppm, and a significant component at around 3.8 ppm, as illustrated in (**d**). Employing 3D multi-voxel spectroscopy with TE = 144 ms, we examined a volume of interest (VOI) encompassing the entire lesion, offering insights into the spatial distribution of metabolites within it. The presence of a consistent peak around 2.0 ppm is noted across all spectra within the lesion. (**f**) showcases one spectrum from a 3D multi-voxel grid (voxel number 18 in (**e**)). Subsequent surgical biopsy confirmed the lesion to be a melanoma metastasis.

In a 58-year-old male, a large necrotic lesion closely related to the dura of the parietal lobe, accompanied by foci of hemorrhage (Figure 4a,b) and displaying a ring-enhancing pattern (Figure 4c), was observed. No significant perilesional edema was detected. On the spectra acquired using the technique with shorter (Figure 4d) and longer (Figure 4f) echo times, a prominent peak around 2 ppm was predominant. Both spectra exhibited a peak at around 3.8 ppm, and the CH2 component of lipids at 1.25 ppm. A 3D MRSI TE = 144 ms and a VOI encompassing the entire lesion (voxel grid shown in Figure 4e) provided the spatial distribution of the metabolites within the lesion. The peak at approximately 2 ppm was present in all spectra within the lesion, with one spectrum from a 3D MRSI grid (voxel number 18 in Figure 4e) shown in Figure 4f. After a surgical biopsy, a melanoma metastasis was confirmed.

### 3.4. Patient 4

**Figure 5:**
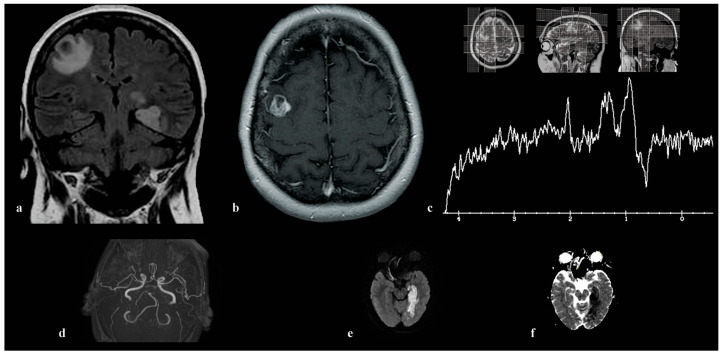
A small, heterogeneous expansive lesion is identified laterally in the right frontal lobe of a 60-year-old female patient. The lesion is shown on the coronal FLAIR image (**a**), exhibiting intense contrast enhancement evident in the axial post-contrast T1 SE image (**b**). Single-voxel MRS with TE = 35 ms reveals a peak around 2.0 ppm, noted as NAA-like, alongside lactate–lipid peaks at 1.3 ppm and a lipid peak at 0.9 ppm (**c**). Following biopsy, metastasis from non-small-cell lung cancer was confirmed. Additionally, a lesion located in the region of the left mesial temporal lobe and medial thalamus was determined to be an acute infarction, resulting from occlusion of the left posterior cerebral artery (PCA) (**d**–**f**). Three-dimensional time-of-flight angiography demonstrates PCA occlusion from the P1 segment (**d**), while diffusion imaging, coupled with the corresponding ADC map, indicates acute ischemic stroke (**e**,**f**).

A small, heterogeneous expansive lesion was identified in the lateral aspect of the right frontal lobe of a 60-year-old female patient with no known primary tumor. This lesion is depicted on the coronal FLAIR image (Figure 5a), and exhibits intense contrast enhancement evident in the axial post-contrast T1 SE image (Figure 5b). SVS MRS, with an echo time of 35 ms, reveals a peak around 2.0 ppm, noted as NAA-like, alongside lactate–lipid peaks at 1.3 ppm and a lipid peak at 0.9 ppm (Figure 5c). Following biopsy, metastasis from non-small-cell lung cancer was confirmed. Additionally, a lesion located in the region of the left mesial temporal lobe and medial thalamus was identified as an acute infarction, resulting from occlusion of the left posterior cerebral artery (PCA) (Figure 5d–f). Three-dimensional time-of-flight angiography demonstrated PCA occlusion from the P1 segment (Figure 5d), while diffusion imaging, coupled with the corresponding ADC map, indicated acute ischemic stroke (Figure 5e,f).

### 3.5. Patient 5

**Figure 6:**
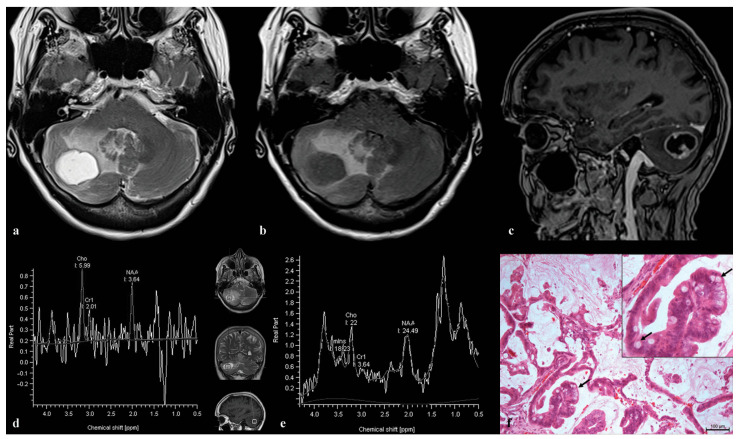
An expansive cystic lesion is observed in the right cerebellar hemisphere of a 55-year-old female patient. The lesion is surrounded by relatively extensive perilesional edema, as depicted on axial T2 (**a**) and FLAIR images (**b**). After contrast administration, enhancement is observed in the soft tissue component, manifested as a thick, irregular wall on the 3D T1 sagittal image (**c**). Single-voxel MR spectra, obtained at 135 ms (**d**) and 30 ms (**e**), show an elevated NAA-like peak around 2.0 ppm, alongside high choline and reduced creatine peaks, as well as lactate and lipid peaks at 1.2–1.3 ppm. The pathohistological specimen (**f**) reveals tumor cells arranged in lepidic and papillary patterns. Goblet cells with abundant intracytoplasmic mucin (arrows) and adjacent alveolar lumens filled with mucin, using H&E × 100, are also observed.

A 55-year-old female presented with headache and progressive ataxia. After a neurological examination, brain MR imaging was performed, revealing an expansive, predominantly cystic lesion in the right cerebellar hemisphere (Figure 6a), with extensive edema (Figure 6b) and post-contrast enhancement of the solid part (Figure 6c). The overall quality of the spectra was low, with a significant amount of noise. Both spectra showed a single peak around 2 ppm (NAA-like), along with increased Cho, low Cr, and clear lipid and lactate peaks at 1.2–1.3 ppm (Figure 6d,e). Since the suspicion for a secondary etiology was raised, lung computerized tomography (CT) was performed, and a biopsy confirmed primary mucinous non-small-cell lung adenocarcinoma (Figure 6f).

## 4. Discussion

In our study, involving 49 participants with confirmed brain metastases, spectra analysis predominantly revealed peaks for Cho and Lip, aligning with the findings of prior studies [10,11,14,15]. Interestingly, an intense peak around 2 ppm, resembling NAA, was observed in five cases. This finding recalls observations from MR spectroscopy in an intracranial sinus mucocele, where a similar peak suggested the presence of N-acetyl mucus compounds, such as N-acetylgalactosamine (GalNAc), N-acetylglucosamine (GlcNAc), N-acetylneuraminic acid (NeuAc), or sialic acid, known constituents of mucin glycoproteins [18]. However, isolating a specific metabolite proved challenging, reflecting the obstacles we encountered in our study. Due to the nature of our in vivo investigation into brain metastases, we were unable to precisely identify the exact metabolite associated with that peak. Although we have confirmation of mucin presence in certain metastases exhibiting an NAA-like peak, it remains uncertain whether this peak results from mucin, and which of its components are responsible. MRS analysis of ovarian tissues unveiled the presence of an NAA-like peak in ovarian mucinous cystadenoma, evident in both its solid and cystic components [19]. This supports the hypothesis that specific metabolites, possibly NAA or N-acetyl groups from glycoproteins, contribute to this resonance [20]. In a broader study of adnexal tumors, an NAA peak was discernable in 67 cases, with a higher intensity noted in malignant tumors, suggesting a potential marker for malignancy [21]. Similar patterns were observed in other cancers, where an NAA-like resonance was linked to the -CH3 moiety of sialic acid or N-acetyl groups of glycoproteins, underscoring the complexity of metabolic changes in cancerous tissues [22,23]. Furthermore, a consistent resonance, at 2.07 ppm across all studied teratomas and in some serous carcinomas, raises questions about the biochemical underpinnings of these observations [24]. In the aforementioned study, encompassing 6 cases of glioblastoma (GBM) and 15 cases of metastases from various primary sites, an unusual narrow peak at 2.05 ppm was observed in 4 lung metastases and a mucocele, using PRESS at TE 30. This peak was attributed to sialic acid, a component of mucin. To validate this hypothesis, spectroscopy on a sialic acid solution within a phantom was conducted. The in vitro spectrum of sialic acid, using PRESS at TE = 30 ms, showed a distinct peak at 2.05 ppm, along with a broader component around 3.8 ppm. The research further revealed that the peak at approximately 2.05 ppm has a longer apparent T2 relaxation time compared to lipid resonances, making it detectable even at extended echo times (136 ms and 272 ms). This attribute raises the possibility of misinterpreting it as the neuronal marker NAA [25]. In our study, lipid peaks in the metastasis of mucinous carcinoma of the breast (Figure 2) decreased significantly, from TE = 35 ms (Figure 2f) to 135 ms (Figure 2e), due to their very short T2 values, unlike the peak around 2 ppm, which showed a lesser influence by echo time. This is one of the advantages of our study, compared to previous ones: we acquired spectra from all patients using both short and long echo times, confirming that the NAA-like peak persists in both. This finding is crucial for ensuring diagnostic protocols do not overlook such features due to technical choices. Furthermore, by applying MRSI, we established the presence of the NAA-like peak throughout the entire tumor, thus ruling out metastasis heterogeneity as a possible cause of this peak appearance. An additional study combining in vitro and in vivo approaches in Salla disease patients suggested that the chemical shifts for NAA and sialic acid in aqueous solution are indistinguishable. It proposed that an elevated concentration of free sialic acid in brain tissue could mimic an increased NAA peak, possibly compensating for the NAA loss in Salla disease [26]. Noteworthy is the detection of high and dominant peaks resembling NAA at 2.0 ppm, higher than those in corresponding normal brain tissues, in two histologically confirmed metastases. These were identified cytologically and immunohistochemically as mucinous adenocarcinomas originating from the biliary and gastric systems. The speculation was that these peaks might be linked to N-acetylglucosamine or galactosamine, components of mucin [27]. Two out of five metastases exhibiting an unusual NAA-like peak in our study were histologically verified to contain a mucinous component: Patient 1, diagnosed with mucinous carcinoma of the breast; and Patient 5, presenting mucinous non-small-cell lung adenocarcinoma. Additionally, our study identified the NAA-like peak in two cases of metastatic melanoma and in one case of metastatic non-small-cell lung cancer. Spectral analysis revealed a prominent peak at approximately 3.8 ppm in two melanoma metastases (Figure 3d and Figure 4d,f), and in one breast cancer metastasis (Figure 2f), suggesting the presence of mucin. In our study, for the first time, a peak around 2 ppm was observed in melanoma metastases. These findings align with evidence indicating the involvement of the mucin protein MUC-1 in melanoma cells, suggesting its role in melanogenesis and metastasis through the modulation of related genes [28], which contrasts with other studies showing that mucin levels are significantly lower in metastatic melanoma compared to non-metastatic form [29]. Sialylation, the enzymatic addition of sialic acid to glycan chains on glycoproteins and glycolipids, is crucial for tumor metastasis, facilitating immune evasion, and promoting migration, invasion, and angiogenesis. The relationship between melanoma progression and altered sialylation pattern, particularly heightened α2,3-sialylation correlating with a more aggressive phenotype, is well documented [30]. Similarly, a link was observed between the intracellular sialic acid levels in breast cancer cells and their metastatic potential [31]. Our findings do not conclusively identify the peak around 2 ppm as sialic acid. Sialic acid has long been recognized for its association with cancer metastasis, with substantial evidence indicating that its accumulation on the cancer cell surface plays a crucial role in promoting cancer migration and survival [32]. The increased sialylation of tumor cells, in contrast to their normal counterparts, has been shown to promote tumor growth and is linked to poorer outcomes for cancer patients [33,34]. There is a need for further investigation to confirm the presence of sialic acid in brain metastases, and to understand its clinical significance fully. Given that sialic acid is a mucin component likely responsible for the 2 ppm peak observed in the metastasis spectra in our study, its detection could serve as a potential marker for adverse prognosis. The presence of sialylation in metastasis could potentially account for the fact that some metastases in our study exhibit an NAA-like peak, while others with the same histopathological diagnosis do not. However, we cannot definitively assert this, due to the lack of mucin testing on all metastases, leaving uncertainty about the mucin content in cases without the 2 ppm peak. This represents a limitation of our study and underscores the need for further research. Alternatively, the signal around 2 ppm may originate from the neurotransmitter N-acetyl-aspartyl-glutamate (NAAG), which has a signal close to the chemical shift observed for NAA. Although NAA is responsible for the largest contribution to the MRS peak at 2 ppm, NAAG can contribute by 10 to 20% to this signal, but very frequently, the entire peak is attributed only to NAA [35,36]. A global metabolomics profiling of ovarian cancer (OVCA) showed that NAAG and NAA levels were more elevated in metastatic OVCA than in primary OVCA or normal ovaries [37]. NAAG is shown to be not only a potential metabolite marker for cancer motoring, but also a glutamate provider that supports cancer growth [38,39]. Our data lack specificity in determining the exact contributors to the 2 ppm peak in brain metastases, necessitating further research. This includes comprehensive histopathology, mucin expression testing, in vitro MRS analyses, and accurate determination of the chemical shifts of the NAA-like peak in metastases. Identifying metastases that express mucin, distinguished from infiltrative tumors typically associated with NAA presence, could significantly refine diagnostic criteria and impact patient management strategies.

## 5. Conclusions

In conclusion, our study contributes to the existing body of knowledge by expanding the spectrum of possible brain metastases expressing mucin, characterized by a prominent NAA-like peak around 2.0 ppm. These findings suggest the potential utility of this diagnostic indicator in identifying individual ring-enhancing lesions as mucin-expressing metastases.

## Figures and Tables

**Table 1:** Patient characteristics.

Origin of Carcinomas	Numbers	Median Age	Female/Male
Lung	18	60.5 (42–79)	8/10
Breast	12	57 (43–71)	12/0
Colorectal	9	60.5 (49–72)	4/5
Melanoma	7	63 (49–77)	2/5
Prostate	2	73 (72–74)	0/2
Kidney	1	67	0/1
Total cohort	49		

## Data Availability

The human data that support the findings of this study are not openly available, due to sensitivity and privacy, and are available from the corresponding author upon request.
